# Influence of Ag-18Cu-10Zn Filler Material on Microstructure and Properties of Laser-Welded Al/Cu Dissimilar Butt Joints

**DOI:** 10.3390/ma17235726

**Published:** 2024-11-22

**Authors:** Ziquan He, Fei Liu, Ping Gao, Lihui Pang, Yong Su

**Affiliations:** School of Materials Science and Engineering, Hefei University of Technology, Hefei 230009, China; 2020217270@mail.hfut.edu.cn (Z.H.); gaoping27388@163.com (P.G.); 13475362582@163.com (L.P.); suyong1963@126.com (Y.S.)

**Keywords:** aluminum, copper, laser welding, microstructure, tensile strength, electrical resistivity

## Abstract

Dissimilar welding between aluminum and copper poses significant challenges, primarily due to differences in their thermal and mechanical properties, resulting in brittle intermetallic compounds, limited joint strength, and high electrical resistivity. This study aims to overcome these issues by employing Ag-18Cu-10Zn filler material and optimizing laser power with a focus on improving joint strength and electrical conductivity. The results indicate that the incorporation of silver and zinc enhances the phase composition and microstructure of the weld. By forming solid solution phases such as Ag_2_Al and Cu_5_Zn_8_, the brittle Al_2_Cu phase commonly found in traditional Al/Cu welding is replaced. This not only promotes the heterogeneous nucleation of fine silver-rich grains but also restricts the excessive growth of silver-poor grains, resulting in a uniform distribution of fine grains throughout the weld. These modifications contribute to both fine-grain strengthening and dispersion strengthening. At an optimal laser power of 750 W, joint strength reaches 109 MPa, while joint resistivity decreases to 3.19 μΩ·cm, 12.6% lower than that of the aluminum alloy base material. This study proposes a process for achieving highly conductive, reliable Al/Cu dissimilar metal joints, potentially impacting the aluminum–copper connections in battery modules for new energy vehicles.

## 1. Introduction

Copper alloys are extensively employed in the electrical and automotive industries due to their superior electrical conductivity, thermal conductivity, and ductility. Aluminum, while exhibiting similar electrical properties to copper, offers the additional advantages of lower cost and higher specific strength. Consequently, aluminum/copper dissimilar metal welding technology has progressed significantly in recent years. In new energy vehicle battery modules, aluminum/copper busbar joints are commonly joined by micro-riveting or mechanical-fastening methods. However, these methods result in increased weight and stress concentrations, and elevated electrical resistivity [1,2]. Therefore, there is a pressing demand for joints that are lightweight and exhibit good electrical conductivity. In microelectronic packaging, aluminum and copper are essential materials for electrical interconnections, and high-quality aluminum/copper joints provide excellent electrical contact performance [3]. Expanding the application of aluminum/copper dissimilar alloy connections will significantly advance related industries.

The differences in thermophysical properties between aluminum and copper, along with the formation of brittle intermetallic compounds (e.g., Al_2_Cu, AlCu, Al_4_Cu_9_), lead to weld areas that are prone to embrittlement and reduced mechanical strength [4,5]. The electrical properties of Al/Cu dissimilar metal joints are particularly crucial. However, these properties are influenced by numerous factors, including weld defects and joint area at the macroscopic level, microstructure morphology, grain size, and intermetallic compounds at the mesoscopic level, as well as metal-ion vibration and carrier reflection or bypassing at the microscopic level. These factors present significant challenges in achieving a reliable connection between aluminum and copper dissimilar metals.

To minimize the formation of Al-Cu intermetallic compounds (IMCs), research has focused on three primary strategies: controlling the welding heat input [3,6,7,8,9,10,11,12,13], reducing the existence and reaction time of the molten pool [14,15,16], and employing metal interlayers to prevent direct contact between Al and Cu. Although the first two strategies can optimize IMC distribution, preventing direct contact between the base materials remains challenging. Under the third strategy, Liu et al. [17] employed FeCoCrNiMn high-entropy alloy powder in laser deposition welding to create a high mixed entropy (ΔS_mix_) environment in both the weld center and the Cu-side weld zone, which facilitated the formation of fine, equiaxed grains. However, a significant amount of Al-Cu intermetallic compounds still formed on the aluminum side of the weld, resulting in a joint tensile strength of only 51 MPa. Yan et al. [18] discovered that Ni could inhibit the diffusion of aluminum and refine the microstructure of the detrimental intermetallic compound (IMC) phase in the interfacial zone. The tensile strength of joints with Ni foils initially reached a maximum of 126.9 MPa. Payak et al. [19] investigated the effect of a Zn interlayer on Al-Cu joints, finding that Zn prevented the formation of a thick intermetallic compound layer between AA6101 and C11000, achieving a maximum strength of 99.84 MPa. Lei et al. [20] explored the effects of pure Al, Al-Si, and Zn-Al brazing materials on the microstructure and mechanical properties of aluminum/copper lap laser brazing. The maximum tensile strengths of joints employing ZnAl_2_, AlSi_12_, and pure aluminum brazing materials were 148 MPa, 109 MPa, and 65 MPa, respectively. Furthermore, materials such as Ag [19,21], Sn [22] Al [23], tin-based alloys [24], and Al-Si alloys [25] have also been employed as intermediate materials to achieve reliable aluminum/copper dissimilar metal connections. As observed above, although the introduction of an intermediate layer can significantly enhance the mechanical properties of Al/Cu joints, the mechanism by which filler metals influence the electrical properties of these joints has been rarely reported. Furthermore, the development of welding consumables to achieve a highly conductive and reliable connection for aluminum/copper dissimilar metals requires further research.

Due to its stability, ductility, and excellent electrical conductivity, silver is often employed as a conductive material. Ag, Al, and Cu can form eutectic compounds in suitable ratios, effectively mitigating the adverse effects of Al-Cu intermetallic compounds (IMCs) on joint properties [26]. Zinc, with its low melting point and high mobility, enhances wettability by promoting the uniform spread of molten metal across the Al/Cu weld when applied to the substrate surface. Given that silver offers better conductivity than copper but slightly lower joint strength, this study employs laser welding technology and designs an Ag-18Cu-10Zn filler material. This study investigates the influence of this filler material at different laser power levels on the weld microstructure, as well as on the mechanical and electrical properties of the joint. The aim is to identify the optimal process conditions that enhance joint strength and electrical properties by using Ag-18Cu-10Zn filler material and optimizing laser power.

## 2. Materials and Methods

**Figure 1:**
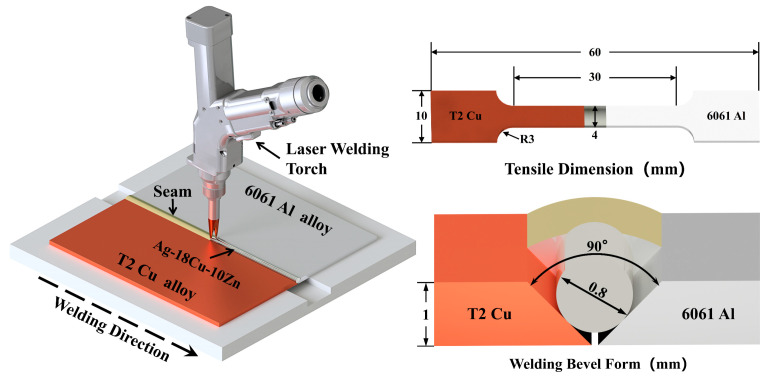
Schematic diagram of welding principle and tensile dimension.

In this experiment, 6061 aluminum alloy from Lihe Metal Materials Co., Ltd., Dongguan City, China and T2 copper plates from Chunshi New Materials Co., Ltd., Taizhou City, China (30 mm × 60 mm × 1 mm) were selected, with their chemical compositions detailed in Table 1. A custom mold was employed to batch-produce 0.8 mm diameter Ag-18Cu-10Zn filler material. A RJ-SC1500 pulsed laser welding machine from Ruiju Electromechanical Equipment Co., Ltd., Shanghai City, China operating at 2000 Hz and 100% duty cycle was employed for laser welding offset by 0.1 mm on the T2 copper surface, using butt-welding configuration with 10 L/min argon shielding gas. The specific welding parameters are listed in Table 2. Tensile tests were conducted according to the ASTM E8/E8M-24 standard [27] on a universal testing machine at a rate of 2 mm/min. The welding principle, groove configuration, and dimensions of the tensile specimens are shown in Figure 1. For metallographic specimen preparation, the samples were cut using a DK7745 EDM wire cutter from Renguang Intelligent Technology Co., Ltd., Suzhou City, China and then mounted using the cold mounting method. The specimen surfaces were rough-ground with sandpaper of gradually increasing grit sizes, from 400 to 1200. The surfaces were then polished using diamond paste, with Keller reagent from Feijing Biotechnology Co., Ltd., Fuzhou City, China (V_HF_:V_HNO3_:V_HCl_:V_H2O_ = 1:1.5:2.5:95) employed for etching the aluminum side and FeCl_3_ solution (FeCl_3_ 1 g, concentrated HCl 20 mL, and H_2_O 100 mL) for the copper side. The weld microstructure and fracture morphology were then observed using SEM3200 from CIQTEK Co., Ltd., Hefei City, China and The XFlash 730M-300 detector from Bruker Corporation, Berlin, Germany was employed for EDS chemical composition analysis. XRD was employed to characterize the phase composition at a voltage of 30 KV, a current of 30 mA and a test angle of 5~90°. Digital microhardness testers were used to perform Vickers hardness tests on the cross-section of metallographic specimens with a load of 200 gf held for 10 s, at intervals ranging from 0.025 mm to 0.25 mm between each test point. Additionally, the resistance of the joints was measured using a GS2540-10A transformer DC resistance tester from Gaosheng Electric Co., Ltd., Yangzhou City, China with Kelvin Four-Wire Method at a current of 10 mA, and the resistivity was then calculated.

## 3. Results and Discussion

### 3.1. Analysis of Surface Appearance of Joint

**Figure 2:**
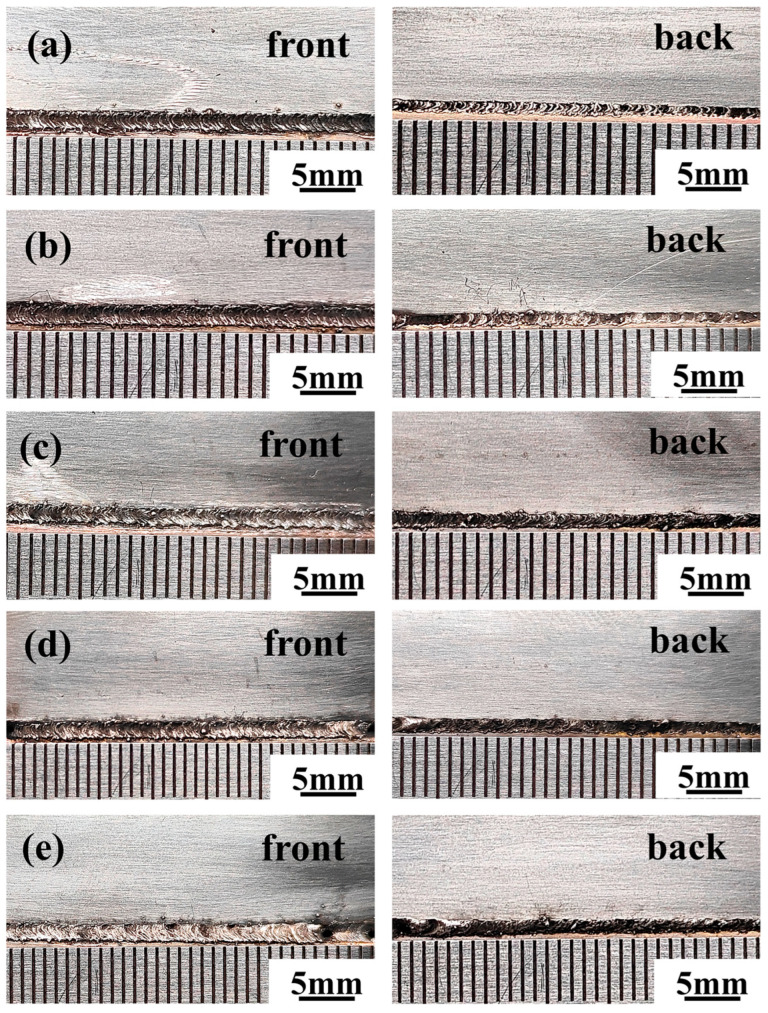
Macro morphology of joints: (**a**) 650 W; (**b**) 700 W; (**c**) 750 W; (**d**) 800 W; (**e**) 850 W.

**Figure 3:**
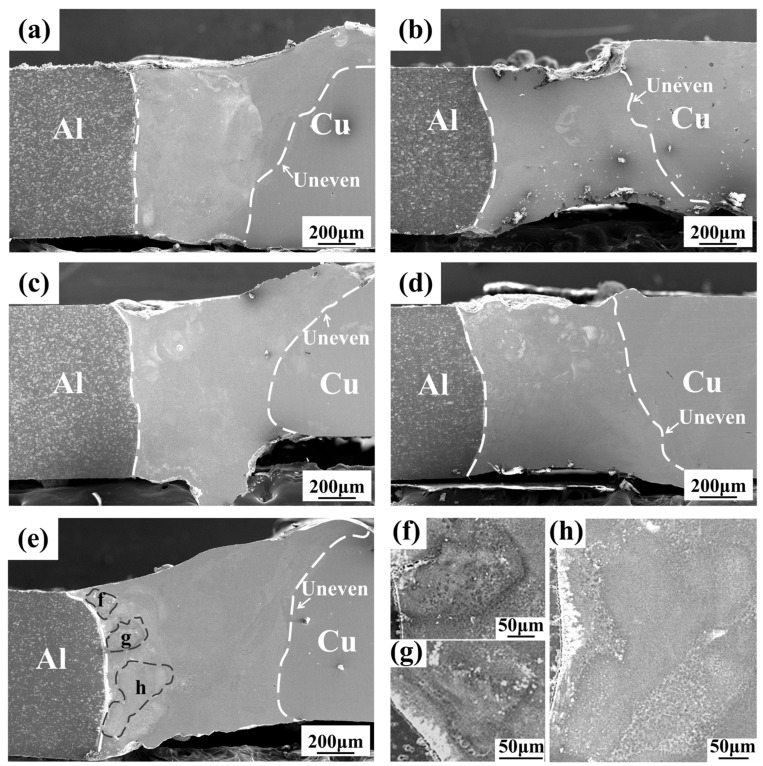
Cross section morphology of joint: (**a**) 650 W; (**b**) 700 W; (**c**) 750 W; (**d**) 800 W; (**e**) 850 W; (**f**–**h**). Enlarged view of three different areas of the weld on the aluminum side of the figure (**e**).

As shown in Figure 2a–e, the overall shape of the joint is satisfactory under different laser powers, with no welding defects such as undercut, spatter, incomplete penetration, lack of fusion, or cracks. Figure 3a–d illustrates that the weld cross-section under various laser powers also shows no significant defects. The interface on the aluminum side appears relatively flat, while the interface on the copper side is irregular. This difference can be attributed to the higher thermal conductivity of the copper base material compared with that of the aluminum base material. Even with laser offset, the copper side experiences less melting, and as a result, the interface retains the beveled shape from before welding. As shown in Figure 3e, three areas with different morphologies appear at the interface front on the aluminum side compared with the center of the weld, as shown in the magnified images in Figure 3f–h. These differences are presumed to result from an increased amount of base metal melting, a longer molten pool duration due to the higher laser power, and a greater diffusion of the alloying elements, leading to a more intense alloying reaction. Figure 3f,g are closer to the heat source during welding and more aluminum elements are involved in the reaction, thus exhibiting a contrast more similar to the aluminum base material compared to Figure 3h.

### 3.2. XRD Phase Analysis of Joints

**Figure 4:**
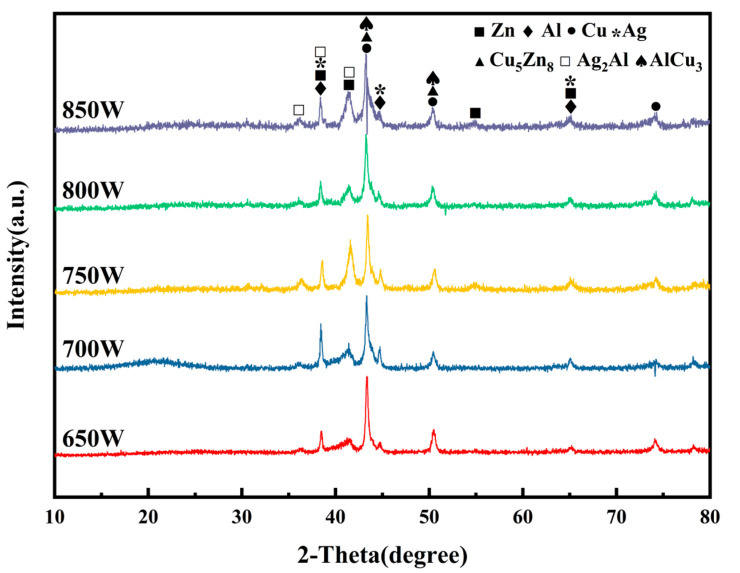
X-ray diffraction pattern of joint.

The X-ray diffraction (XRD) patterns in Figure 4 show that the primary phases in the weld remain consistent across different laser power levels. Solid solutions of aluminum, copper, silver, and zinc, as well as the intermetallic compounds AlCu_3_, Ag_2_Al, and Cu_5_Zn_8_, are consistently detected. Therefore, variations in laser power do not affect the types of main phases present in the weld. The diffraction pattern indicates a slight increase in the content of the Zn-based solid solution and Ag_2_Al in the fusion zone when the laser power is set to 750 W. Additionally, as the laser power increases, the content of AlCu_3_ intermetallic compounds in the weld gradually rises.

### 3.3. Analysis of Microstructure of Joint

#### 3.3.1. Microstructure of Aluminum Side Weld

**Figure 5:**
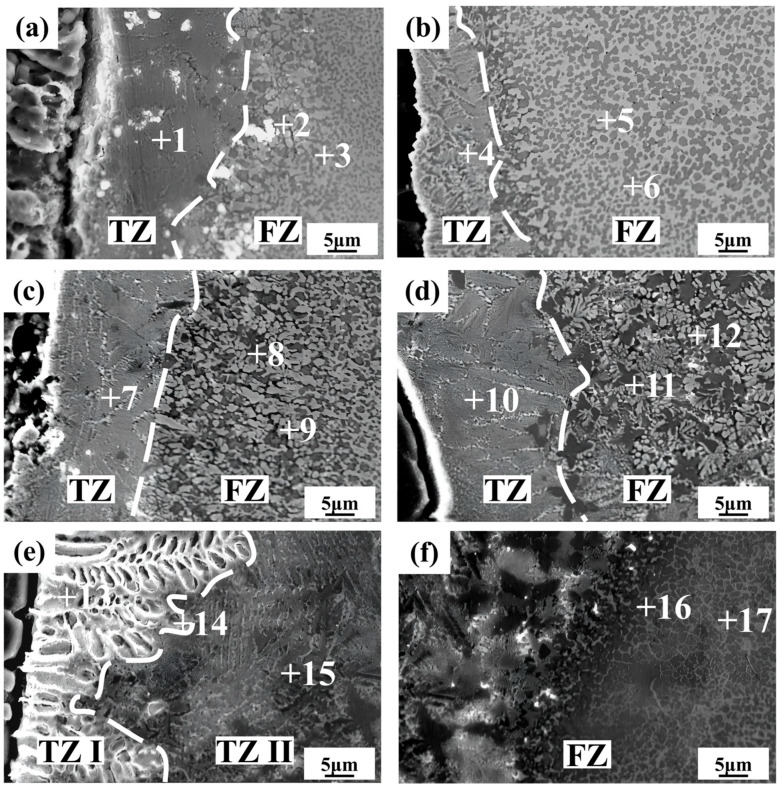
SEM images of microstructure of aluminum side weld in SE mode: (**a**) 650 W; (**b**) 700 W; (**c**) 750 W; (**d**) 800 W; (**e**) and (**f**) 850 W.

As shown in Figure 5a–e, a transition zone (TZ) exists between the aluminum alloy and the weld, and its width increases with the rise in laser power. The gray microstructure of the TZ is densely distributed but irregularly oriented, and the grain size of the columnar crystals is larger, resembling a feather-like morphology. The fusion zone (FZ) consists of black and white grains. Figure 5a shows that the TZ width is inconsistent, suggesting that the energy provided by the 650 W laser power is insufficient and unstable, leading to inadequate elemental diffusion and insufficient molten pool stirring, resulting in heterogeneous weld microstructure. Figure 5b shows that the increased laser power leads to homogenization of the TZ, with an overall width of 13 μm and a slight increase in the size of black grains in the FZ at 700 W. As shown in Figure 5c,d, from 750 W to 800 W, the increased heat input enhances elemental diffusion at higher temperatures, accelerating the migration of aluminum atoms from the base material into the molten pool, which widens the transition zone. The grain growth rate in the FZ also increases with the higher welding energy and extended molten pool retention time, resulting in the coarsening of black and white grains, transitioning from equiaxed grains and network structures to dendritic and irregularly oriented columnar crystals. As shown in Figure 5e, two distinct transition zones, TZ I and TZ II, are observed at 850 W. The morphology of TZ I differs from those at other laser powers, displaying coarse dendritic crystals oriented perpendicularly to the base material interface, with varying dendrite widths. Compared with point 15 in TZ II, point 13 in TZ I shows significantly higher levels of zinc and silver and lower levels of copper. This may be due to the long-range diffusion of zinc, silver, and copper atoms, facilitated by sufficient welding energy, converging in TZ I along with a large number of molten aluminum atoms from the base material. The larger temperature gradient promotes the nucleation and growth of coarse white dendrites of Zn, Ag, and Al elements, while Cu elements are rejected to the solid–liquid interface frontiers and segregate, forming TZ II in combination with other elements. This also explains why TZ II exhibits a similar morphology to other laser powers, but with higher copper content at point 15. Figure 5f shows the fusion zone at 850 W. The size of the black silver-poor grains further increases with irregular distribution, and the morphology of the white silver-rich grains becomes reticular. This microstructure distribution negatively affects the mechanical properties of the joint.

Combining the XRD diffraction results Figure 4, the possible phase compositions and EDS elemental composition analysis of various microstructures in the central region of the weld are summarized in Table 3. Points 1, 4, 7, 10, and 15 correspond to the gray microstructure, which primarily forms from the reaction between Ag, Al, and Cu, resulting in Ag_2_Al, AlCu_3_, and an Al-based solid solution, with a relatively small amount of Zn that reacts with Cu to produce a minor quantity of Cu_5_Zn_8_. Points 2, 6, 8, and 11 correspond to the silver-poor black grains in the FZ, which contain relatively small amounts of Zn and Ag, primarily forming the AlCu_3_ intermetallic compound. Points 3, 5, 9, and 12 represent the silver-rich white microstructure, where Ag_2_Al intermetallic compounds and an Al-based solid solution are mainly generated, with small amounts of copper and zinc reacting to form Cu_5_Zn_8_.

#### 3.3.2. Microstructure of Weld Center

**Figure 6:**
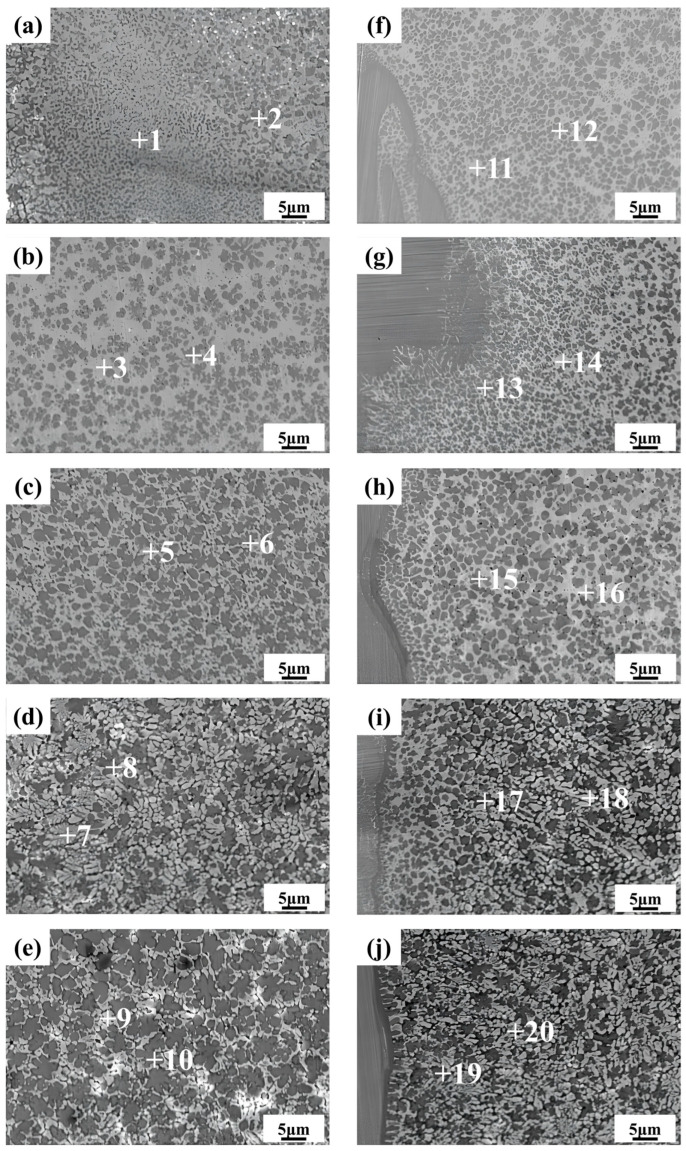
SEM images of microstructure of weld center in SE mode: (**a**) 650 W; (**b**) 700 W; (**c**) 750 W; (**d**) 800 W; (**e**) 850 W and microstructure of copper side weld area in SE mode: (**f**) 650 W; (**g**) 700 W; (**h**) 750 W; (**i**) 800 W; (**j**) 850 W.

As shown in Figure 6a–e, similar to the FZ on the aluminum side, black and white microstructures appear in the center of the welds. With the increase in laser power, the refinement effect of the white grains on the black grains gradually weakens, leading to an increase in the size of the black grains, evolving from small spot-like grains to coarse dendritic structures. Simultaneously, as the black grains grow, the zinc and silver content in their composition decreases, as indicated by points 2, 4, 6, 8, and 10. This suggests that the zinc and silver content has a significant effect on the refinement of the black grains. At lower power levels, these elements promote the nucleation of black grains and inhibit their growth. Figure 6e shows that the black grains contain a substantial amount of hard and brittle aluminum–copper intermetallic compounds, which significantly degrade the mechanical properties of the weld center’s microstructure, potentially leading to joint fracture during tensile testing, as shown in Section 3.4.3. Combining the XRD diffraction results (Figure 4), the possible phase compositions and EDS elemental composition analysis of various microstructures in the weld center region are summarized in Table 4. The white grains are silver-rich phases, primarily consisting of (Al) + Ag_2_Al + Cu_5_Zn_8_, while the black grains are silver-poor phases, primarily consisting of (Al) + AlCu_3_.

#### 3.3.3. Microstructure of Copper Side Weld

As shown in Figure 6f–j, the size of the black grains increases only slightly with rising laser power, while the white grains transition from a reticulated structure to a more granular and striated morphology. As indicated by points 8 and 10, the copper and zinc contents in the white grains shown in Figure 6i,j is even lower compared with those at lower power, while the aluminum content is higher. This is due to the increased laser power, which facilitates the diffusion of a large amount of aluminum into the copper side, while the zinc content remains limited. According to the Zn-Al binary phase diagram, zinc atoms do not form intermetallic compounds with aluminum, and their solid solubility does not exceed 5% [28]. The decrease in copper content is attributed to the formation of the intermetallic compound AlCu_3_ between the excess aluminum and copper in the black grains. In the silver-rich white grains, the reduced copper content helps to minimize the formation of aluminum–copper intermetallic compounds, while a moderate amount of zinc and a small amount of copper contribute to the formation of limited amounts of Cu_5_Zn_8_ intermetallic compounds. Cu_5_Zn_8_ serves as a nucleation core, promoting the nucleation of Ag_2_Al and the refinement of the aluminum-based solid solution, thereby achieving fine-grain strengthening in the white phases. The extensive nucleation of white grains at higher laser power restricts the growth of black grains, which are confined to the limited space, significantly reducing the number of black grains that appear as coarse dendritic crystals. Additionally, the grain size near the copper base metal interface is smaller than that near the weld, owing to the faster heat transfer in the molten pool near the copper base metal, which facilitates heat dissipation, resulting in smaller cellular black grains. The weld on the copper side exhibits a more uniform microstructure with finer grains and fewer black grains, making it less likely to be the preferred fracture zone. Based on the XRD diffraction results in Figure 4, the possible phase compositions and EDS elemental composition analysis of the various microstructures in the copper-side weld region are summarized in Table 4. The white grains are silver-rich phases, consisting primarily of (Al) + Ag_2_Al + Cu_5_Zn_8_ or (Ag) + Ag_2_Al + Cu_5_Zn_8_, while the black grains are silver-poor phases, primarily composed of (Al) + AlCu_3_.

### 3.4. Properties and Fracture Analysis of Welded Joints

#### 3.4.1. Analysis of Mechanical Properties of Joints

**Figure 7:**
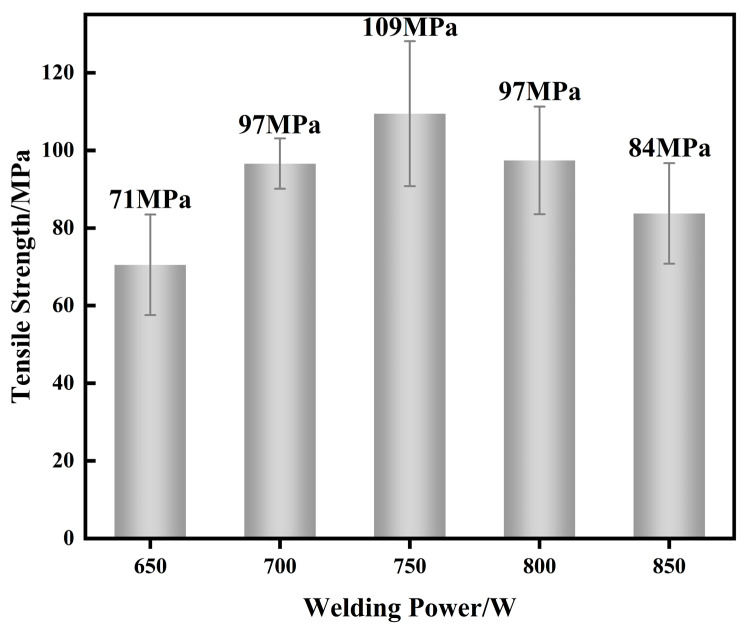
Tensile strength of joint under different laser power.

As shown in Figure 7, with increasing laser power, the tensile strength first increases and then decreases. At 750 W, the mechanical properties are optimal, with an average tensile strength of 109 MPa and a maximum single tensile strength of 134 MPa, which is higher than that of Al/Cu dissimilar alloy joints with pure silver and zinc filler materials [19,20,21]. This indicates that the addition of the Ag-18Cu-10Zn alloy as an intermediate layer significantly enhances the mechanical properties of the aluminum/copper dissimilar alloy joints. Figure 3b shows that the melting volume of the base metal and the melting rate of the weld metal are lower due to the lower laser power, resulting in a smaller effective joint area at laser powers ranging from 650 W to 700 W. Figure 5a and Figure 6f show that the hydrodynamic stirring effect of the molten pool under lower laser power is insufficient, leading to discontinuities in the joint microstructure. Additionally, the low boiling point of zinc in the filler material causes zinc vapors to escape from the weld too late due to the short duration of the molten pool, leading to the formation of small pores and weld defects. As a result, despite the smaller number of black phases (primarily Al/Cu intermetallic compounds) and the thinner transition zone (TZ), the joints still exhibit relatively low mechanical properties at lower power. At 750 W, these issues are mitigated. The addition of silver and zinc contributes to the fine-grain strengthening of the black grains and the diffuse strengthening effect of the fine white grains in the aluminum-based and silver-based solid solutions. The reduction in the formation of aluminum–copper intermetallic compounds, along with the thinner transition zone on the aluminum side, further enhanced the mechanical properties of the weld. These factors result in high overall tensile strength, with failure occurring in the relatively weak interface between FZ and TZ. At 800–850 W, the mechanical properties deteriorate due to the further widening of the transition zone on the aluminum side and the increasing size of the black grains in both the fusion zone and the weld center.

#### 3.4.2. Analysis of Hardness Distribution of Joints

**Figure 8:**
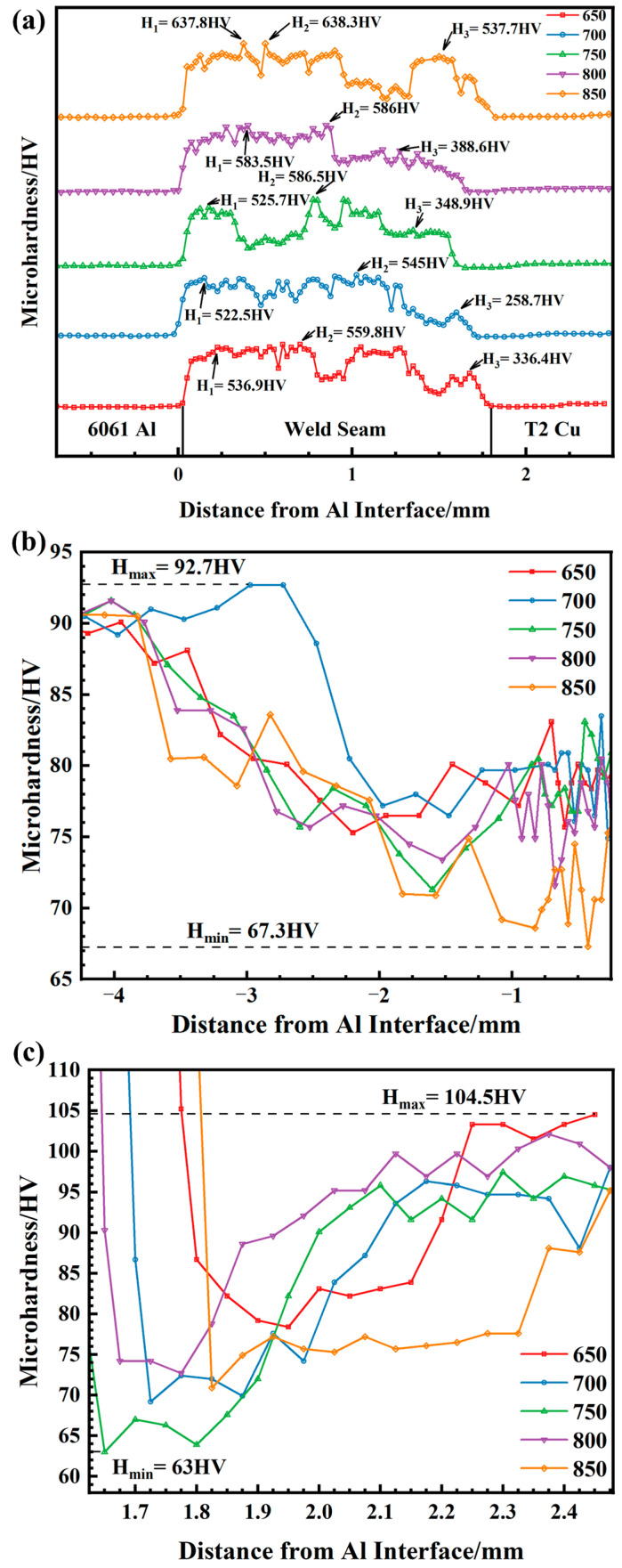
Microhardness distribution of joint under different laser power: (**a**) weld center. H_1_, H_2_, and H_3_ represent the maximum hardness values of the aluminum side weld, center weld, and copper side weld, respectively; (**b**) aluminum side; (**c**) copper side.

As shown in Figure 8a, the weld hardness on the copper side is significantly lower than on the aluminum side. The weld hardness reaches its peak at 850 W, which is attributed to the high heat input generating a large number of intermetallic compounds (e.g., Ag_2_Al, AlCu_3_). The fluctuating hardness in the weld center is due to the coexistence of softer white grains and harder black grains. The weld zone on the aluminum side exhibits a stable hardness, which helps to reduce residual stresses, achieve uniform stress distribution, and lower the risk of brittle fracture. Figure 8b,c illustrates that the base material softens under different laser powers. In the heat-affected zone (HAZ) of the Cu base material, the Vickers hardness ranges from 63 HV to 104.5 HV, with a reduction of about 40 HV, while in the HAZ of the Al base material, the Vickers hardness ranges from 67.3 HV to 92.7 HV, with a reduction of about 25 HV. However, the width of the softened zone in the base material is not affected by laser power, indicating that the Ag-18Cu-10Zn filler material can effectively stabilize and control the weak areas of the joint’s mechanical properties, mitigating the softening phenomenon.

#### 3.4.3. Fracture Analysis of Joints

**Figure 9:**
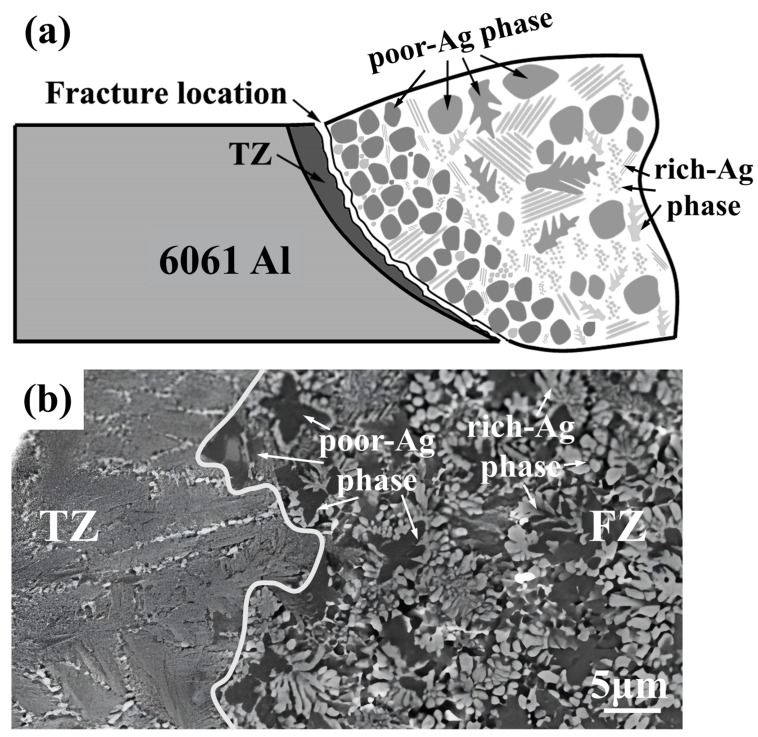
Fracture reason of the joint: (**a**) schematic of the fracture; (**b**) the microstructure of the transition zone and the fusion zone.

Figure 9a shows the schematic of the fracture location. Due to the improved microstructure of the FZ, the performance of the joint was enhanced, and the interface between the TZ and the FZ became a new weak part of the joint, as shown in Figure 9b. The reason may be that the structure between these two parts is obviously different, and there is a large area of black silver-poor grains aggregating at the interface, which is prone to stress concentration during stressing.

**Figure 10:**
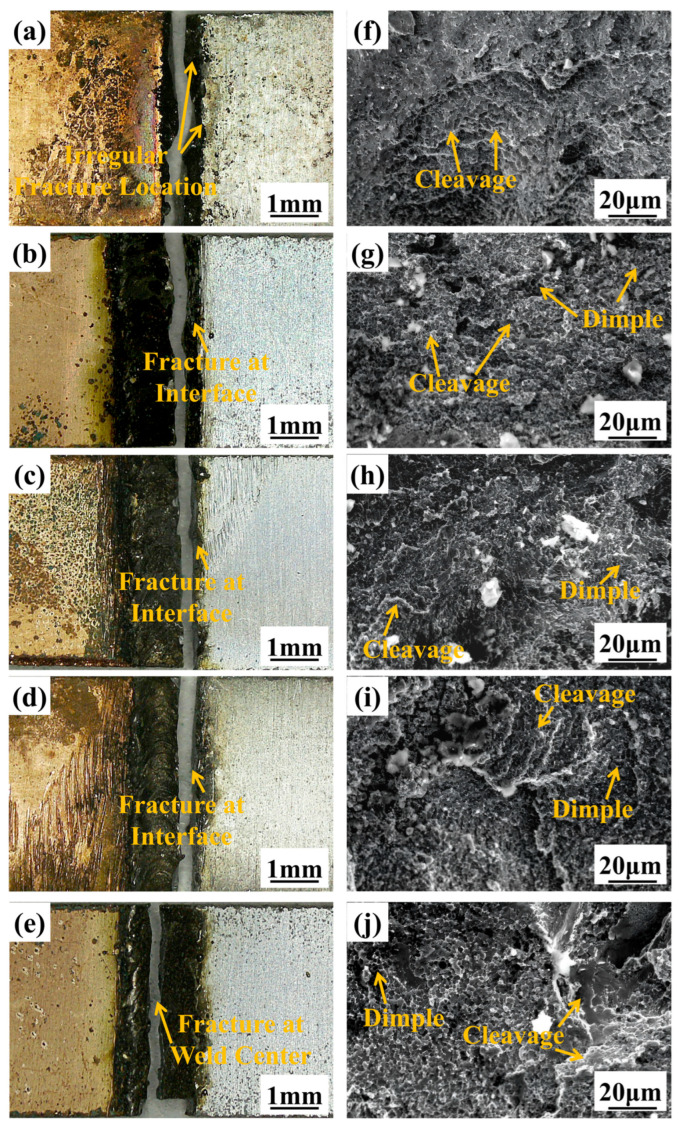
Fracture morphology of joint under different laser power: Macrostructure (**a**) 650 W; (**b**) 700 W; (**c**) 750 W; (**d**) 800 W; (**e**) 850 W; Microstructure (**f**) 650 W; (**g**) 700 W; (**h**) 750 W; (**i**) 800 W; (**j**) 850 W.

As shown in Figure 10a, the location of the weld fracture is not fixed, with cracks extending from the aluminum side through the weld center to the copper side. This is due to the non-uniform distribution of elements and composition in the weld at this power level, which leads to an irregular distribution of brittle and hard phases, resulting in stress concentrations at multiple points. As observed in Figure 10f, the fracture near the weld center exhibits flat pseudo-cleavage planes, dimples, and plastic tearing edges, indicating a ductile–brittle mixed fracture. The fracture locations in Figure 10b–d are in the interface between the TZ and the FZ, while in Figure 10e, the fracture occurs in the weld center. Based on the mechanical property test results, it can be inferred that the mechanical properties of the joint are better when the fracture occurs in the interface. Observations from Figure 10g–j show that the fracture mechanism of the joint remains a ductile–brittle mixed fracture under laser powers ranging from 700 W to 850 W.

#### 3.4.4. The Mechanism of the Role of Filler Material

As shown in Figure 9a, in terms of the phases in the weld, the addition of silver and zinc has a positive effect. Silver replaces copper on the aluminum side, reacting with aluminum to form Ag_2_Al and aluminum-based solid solutions, while on the copper side, it forms a silver–copper eutectic with copper [19]. These intermetallic compounds replace the brittle Al_2_Cu phase commonly formed in conventional Al/Cu welding, enhancing plasticity and electrical conductivity. The introduction of zinc partially inhibits the formation of brittle Al-Cu intermetallic compounds, as zinc preferentially combines with copper to form Zn-Cu compounds (e.g., Cu_5_Zn_8_), reducing the amount of brittle Al-Cu phase in the weld. Compared with Al-Cu compounds, Zn-Cu compounds exhibit greater toughness and ductility, which improves the mechanical properties of the welded joints [20]. As a result, the primary phases in the weld transition from conventional Al-Cu IMCs to Al-based and Cu-based solid solutions, Ag_2_Al, Cu_5_Zn_8_, and a small amount of AlCu_3_, further enhancing the mechanical strength and electrical conductivity of the joints.

From a microstructural perspective, the addition of silver and zinc promotes nucleation, facilitating the heterogeneous nucleation of white, silver-rich grains while inhibiting the excessive growth of silver-poor black grains, thereby refining the grain size. Silver generates fine white silver-rich and black silver-poor grains that are uniformly distributed throughout the weld seam, effectively preventing the segregation and agglomeration of black grains [21]. The low melting point of zinc allows it to diffuse evenly in the weld zone, forming uniform, thinner, and more ductile white grains at the aluminum–copper interface, avoiding the buildup of thick, brittle Al-Cu compounds. Additionally, the formation of white silver-rich grains regulates the local copper content, inhibiting the growth of black-grain dendrites and reducing stress concentrations. The combined addition of silver and zinc enables both fine grain strengthening and dispersion strengthening through diffusely distributed secondary phase particles, which comprehensively improves the mechanical properties of the weld.

#### 3.4.5. Analysis of Electrical Conductivity of Joints

**Figure 11:**
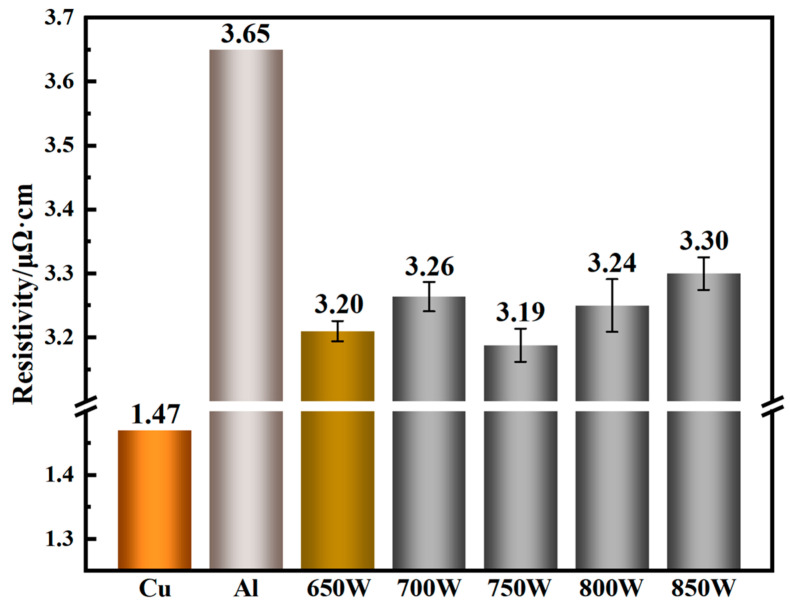
Resistivity of base metal and joint under different laser power.

The resistivity of intermetallic compounds is typically an order of magnitude higher than that of the base material, making intermetallic compounds one of the main factors affecting the electrical conductivity of the joints [29]. As shown in Figure 11, with increasing power, the resistivity of the joints obtained at 650–750 W shows no significant trend in change. The difference in electrical properties between the joints produced at 650 W and 750 W is relatively small. This can be attributed to the fact that, although fewer intermetallic compounds form at 650 W, the presence of pores and other weld defects, as well as the inhomogeneous microstructure, negatively affects conductivity. In contrast, at 750 W, not only is the formation of intermetallic compounds limited, but no other factors degrade the conductivity, resulting in similar resistivity values at both 650 W and 750 W, despite the expectation of lower resistivity at 650 W. The sudden increase in resistivity at 700 W may result from the smaller cross-sectional area of the joint, as shown in Figure 3b, which prevents the cross-section from matching that of the base material, thus increasing the error in resistivity calculation. With a further increase in laser power, the increase in intermetallic compounds in the weld, the larger grain size, and the potential softening of the base material contribute to the rise in resistivity, reaching a maximum of 3.30 μΩ·cm. The aluminum/copper dissimilar metal welded joints produced using Ag-18Cu-10Zn filler material exhibit favorable resistivity, ranging from 3.19 to 3.30 μΩ·cm. Compared with the resistivity of the 6061 aluminum alloy base material (3.65 μΩ·cm), the resistivity of the joints is reduced by up to 12.6%.

## 4. Conclusions

In this study, laser welding technology was employed to investigate the regulation and influence of Ag-18Cu-10Zn filler material on the microstructure, mechanical, and electrical properties of the weld under different laser powers. The following conclusions can be drawn:

Aluminum/copper dissimilar alloy butt welding using 0.8 mm Ag-18Cu-10Zn filler material results in well-formed joints with no obvious defects. The primary phases present in the weld at different power levels include solid solutions of Al, Ag, Cu, and Zn, as well as intermetallic compounds such as AlCu_3_, Ag_2_Al, and Cu_5_Zn_8_. The incorporation of silver and zinc enhances the phase composition and microstructure of the weld. By forming solid solution phases such as Ag_2_Al and Cu_5_Zn_8_, the brittle Al_2_Cu phase commonly found in traditional Al/Cu welding is replaced. This not only promotes the heterogeneous nucleation of fine silver-rich grains but also restricts the excessive growth of silver-poor grains, resulting in a uniform distribution of fine grains throughout the weld. These modifications contribute to both fine-grain strengthening and dispersion strengthening.

As laser power increases, the width of the transition zone (TZ) on the aluminum side shows an increasing trend. The gray microstructure in the transition zone is densely distributed but irregularly oriented, with larger columnar grains exhibiting a feather-like morphology. The tensile strength of the joints first increases and then decreases with increasing laser power. A maximum tensile strength of 109 MPa and a maximum single tensile strength of 134 MPa is reached at 750 W. Fracture occurs in the interface between the TZ and the FZ, where the mechanical properties exhibit a ductile–brittle mixed fracture. The resistivity of the joints shows an overall increasing trend, ranging from 3.19 to 3.30 μΩ·cm, with a maximum decrease of 12.6% compared with the 6061 aluminum alloy base material.

This Ag-18Cu-10Zn filler material has potential applications such as electric vehicles battery module busbar and electronic components, where its ability to enhance mechanical strength and electrical conductivity in lightweight Al/Cu joints can significantly improve efficiency and reliability.

## Figures and Tables

**Table 1:** Chemical composition of base materials (Mass Fraction, wt.%).

Base Materials	Chemical Composition								
Al 6061	Si	Fe	Cu	Mn	Mg	Cr	Zn	Ti	Al
	0.65~0.80	0.7	0.15~0.20	0.15	0.8~0.9	0.08~0.12	0.25	0.15	Bal.
Cu T2	Zn	Sn	Ni	S	Pb	O	Fe	As	Cu
	0.002~0.003	0.002	0.003~0.005	0.005	0.005	0.004~0.006	0.006	0.002	≥99.99

**Table 2:** Laser welding parameters.

Laser Power/W	650	700	750	800	850
Welding speed/(mm·s^−1^)	13	13	13	13	13
Defocus amount/mm	0	0	0	0	0
Pulse frequency/Hz	50	50	50	50	50

**Table 3:** Chemical composition of the measured points in Figure 5 by EDS analysis.

Point	Al (at%)	Cu (at%)	Zn (at%)	Ag (at%)	Possible Phase
1	46.54	24.06	8.34	21.06	(Al) + AlCu_3_ + Ag_2_Al + Cu_5_Zn_8_
2	51.72	38.39	4.24	5.65	(Al) + AlCu_3_
3	31.33	8.38	15.66	44.63	(Al) + Ag_2_Al + Cu_5_Zn_8_
4	47.03	25.58	6.31	21.08	(Al) + AlCu_3_ + Ag_2_Al + Cu_5_Zn_8_
5	30.20	14.70	14.92	40.18	(Al) + Ag_2_Al + Cu_5_Zn_8_
6	47.19	43.87	5.23	3.71	(Al) + AlCu_3_
7	49.40	18.17	9.00	23.43	(Al) + AlCu_3_ + Ag_2_Al + Cu_5_Zn_8_
8	58.65	35.99	2.78	2.58	(Al) + AlCu_3_
9	36.54	14.76	13.20	35.50	(Al) + Ag_2_Al + Cu_5_Zn_8_
10	47.89	23.26	6.56	22.28	(Al) + AlCu_3_ + Ag_2_Al + Cu_5_Zn_8_
11	53.99	33.46	4.63	7.92	(Al) + AlCu_3_
12	45.57	16.49	10.65	27.29	(Al) + Ag_2_Al + Cu_5_Zn_8_
13	44.26	13.62	10.73	31.39	(Al) + Ag_2_Al + Cu_5_Zn_8_
14	49.02	18.32	7.61	25.06	(Al) + AlCu_3_ + Ag_2_Al + Cu_5_Zn_8_
15	56.47	30.56	2.11	10.86	(Al) + AlCu_3_ + Ag_2_Al + Cu_5_Zn_8_
16	44.81	45.41	4.54	5.24	(Al) + AlCu_3_
17	30.13	22.98	12.90	33.99	(Al) + Ag_2_Al + Cu_5_Zn_8_

**Table 4:** Chemical composition of the measured points in Figure 6 by EDS analysis.

Point	Al (at%)	Cu (at%)	Zn (at%)	Ag (at%)	Possible Phase
1	24.84	21.69	17.88	35.59	(Al) + Ag_2_Al + Cu_5_Zn_8_
2	42.04	42.04	6.38	9.53	(Al) + AlCu_3_
3	21.90	11.07	18.67	48.37	(Ag) + Ag_2_Al + Cu_5_Zn_8_
4	30.47	53.78	6.45	9.31	(Al) + AlCu_3_
5	33.53	13.55	14.40	38.52	(Al) + Ag_2_Al + Cu_5_Zn_8_
6	42.60	47.33	5.44	4.63	(Al) + AlCu_3_
7	43.39	21.78	9.01	25.82	(Al) + Ag_2_Al + Cu_5_Zn_8_
8	51.36	41.59	4.00	3.05	(Al) + AlCu_3_
9	37.28	13.77	12.56	36.39	(Al) + Ag_2_Al + Cu_5_Zn_8_
10	48.87	45.01	3.45	2.66	(Al) + AlCu_3_
11	9.23	19.75	19.42	51.60	(Ag) + Ag_2_Al + Cu_5_Zn_8_
12	24.94	59.10	8.55	7.41	(Al) + AlCu_3_
13	26.30	56.90	7.94	8.86	(Al) + AlCu_3_
14	20.60	14.42	18.12	46.85	(Ag) + Ag_2_Al + Cu_5_Zn_8_
15	32.12	53.06	5.47	9.35	(Al) + AlCu_3_
16	26.07	10.70	17.71	45.52	(Ag) + Ag_2_Al + Cu_5_Zn_8_
17	52.31	38.68	3.21	5.81	(Al) + AlCu_3_
18	33.56	8.07	14.69	43.68	(Al) + Ag_2_Al + Cu_5_Zn_8_
19	50.32	41.18	3.98	4.52	(Al) + AlCu_3_
20	35.78	7.81	10.98	45.42	(Al) + Ag_2_Al + Cu_5_Zn_8_

## Data Availability

The original contributions presented in the study are included in the article, further inquiries can be directed to the corresponding author.

## References

[1] Li J., Zillner J., Balle F. (2023). In-depth evaluation of ultrasonically welded Al/Cu joint: Plastic deformation, microstructural evolution, and correlation with mechanical properties. Materials.

[2] Kaufmann F., Strugulea M., Höltgen C., Roth S., Schmidt M. (2023). Seam properties of overlap welding strategies from copper to aluminum using green laser radiation for battery tab connections in electric vehicles. Materials.

[3] Shin W.-S., Cho D.-W., Jung D., Kang H., Kim J.O., Kim Y.-J., Park C. (2021). Investigation on laser welding of Al ribbon to Cu sheet: Weldability, microstructure, and mechanical and electrical properties. Metals.

[4] Beygi R., Carbas R., Marques E., Barbosa A., Kasaei M., da Silva L. (2024). Mechanism of toughness enhancement of brittle fracture by intermittent η-intermetallic in Al/Cu joint made by FSW. Mater. Sci. Eng. A.

[5] Yan S., Li Z., Song L., Zhang Y., Wei S. (2023). Research and development status of laser micro-welding of aluminum-copper dissimilar metals: A review. Opt. Lasers Eng..

[6] Dai W., Guo W., Li Q., Xiao J., Li W., Zhang H. (2024). Homogenization of local microstructure and mechanical properties in friction stir welded Al-Cu alloy joint achieved through laser shock peening. J. Mater. Process. Technol..

[7] Milašinović V., Alil A., Milašinović M., Vencl A., Hatala M., Dikić S., Gligorijević B. (2024). Continuous drive friction welded Al/Cu joints produced using short welding time, elevated rotational speed, and high welding pressures. Materials.

[8] Ma B., Gao X., Huang Y., Zhang Y., Huang Y. (2024). Effect of different pulse shapes on the laser welding of aluminum and copper. Opt. Laser Technol..

[9] Li G., Song J., Lu X., Zhu X., Xu S., Guo Y. (2020). Investigation on microstructure and mechanical properties of Al/Cu butt joints by CMT method in asymmetrical V-groove configuration. Metall. Res. Technol..

[10] Khajeh R., Jafarian H.R., Seyedein S.H., Jabraeili R., Eivani A.R., Park N., Kim Y., Heidarzadeh A. (2021). Microstructure, mechanical and electrical properties of dissimilar friction stir welded 2024 aluminum alloy and copper joints. J. Mater. Res. Technol..

[11] Chang Z., Huang M., Wang X., Wang H., Sun G., Zhou L. (2023). Microstructure Evolution and Mechanical Properties of Thick 2219 Aluminum Alloy Welded Joints by Electron-Beam Welding. Materials.

[12] Kumar N., Masters I., Das A. (2021). In-depth evaluation of laser-welded similar and dissimilar material tab-to-busbar electrical interconnects for electric vehicle battery pack. J. Manuf. Process..

[13] Vatnalmath M., Auradi V., Murthy B.V., Nagaral M., Pandian A.A., Islam S., Khan M.S., Anjinappa C., Razak A. (2023). Impact of bonding temperature on microstructure, mechanical, and fracture behaviors of TLP bonded joints of Al2219 with a Cu interlayer. ACS Omega.

[14] Sas-Boca I.-M., Iluțiu-Varvara D.-A., Tintelecan M., Aciu C., Frunzӑ D.I., Popa F. (2022). Studies on Hot-Rolling Bonding of the Al-Cu Bimetallic Composite. Materials.

[15] Tayebi P., Nasirin A.R., Akbari H., Hashemi R. (2024). Experimental and Numerical Investigation of Forming Limit Diagrams during Single Point Incremental Forming for Al/Cu Bimetallic Sheets. Metals.

[16] Goodarzi N., Hashemi R., Abedini R. (2024). Microstructure investigation and optimization of process parameters of ultrasonic welding for Al–Cu dissimilar joints using design of experiment. J. Mater. Res. Technol..

[17] Liu D., Ni C., Ma Z., Li B., Tang Y., Wang X. (2024). Microstructure and mechanical properties of dissimilar metal joints of copper/aluminum using FeCoCrNiMn filler material. Weld. World.

[18] Yan S., Shi Y. (2020). Influence of Ni interlayer on microstructure and mechanical properties of laser welded joint of Al/Cu bimetal. J. Manuf. Process..

[19] Payak V., Paulraj J., Roy B.S., Bhargava M., Choudhury S. (2023). Microstructural and mechanical characteristics of friction stir welded Al6101/C11000 joints with zinc and silver interlayer. Int. J. Adv. Manuf. Technol..

[20] Lei Z., Zhang X., Liu J., Li P. (2021). Interfacial microstructure and reaction mechanism with various weld fillers on laser welding-brazing of Al/Cu lap joint. J. Manuf. Process..

[21] Chen T., Liu F., Pang L., Hu H., Gao P. (2024). Microstructure and performance study of Al/Cu Laser welding with Ag interlayer. Int. J. Precis. Eng. Manuf..

[22] Eslami N., Hischer Y., Harms A., Lauterbach D., Böhm S. (2019). Influence of copper-sided tin coating on the weldability and formation of friction stir welded aluminum-copper-joints. Metals.

[23] Guo J., Li C., Bian J., Zhang J., Geng B. (2023). Microstructures and Electrical Resistivity of Aluminum–Copper Joints. Metals.

[24] Huan P.-C., Tang X.-X., Sun Q., Akira K., Wang X.-N., Wang J., Wang J.-L., Wei X., Di H.-S. (2022). Comparative study of solder wettability on aluminum substrate and microstructure-properties of Cu-based component/aluminum laser soldering joint. Mater. Des..

[25] Xu W., Yang J., Peng M., Zhao Y., Liu H., Deng P., Gao Y., Zhang H. (2023). Characteristics of torch-offset cold metal transition-cycle step welding of 5052Al alloy with T2 copper via Al-12Si filler. J. Mater. Eng. Perform..

[26] Witusiewicz V., Hecht U., Fries S., Rex S. (2005). The Ag–Al–Cu system: II. A thermodynamic evaluation of the ternary system. J. Alloys Compd..

[27] (2024). Standard Test Methods for Tension Testing of Metallic Materials.

[28] Zhang Z., Zhang J., Zhao X., Cheng X., Liu X., Zhang Q. (2024). Thermodynamic Simulation Calculations of Phase Transformations in Low-Aluminum Zn-Al-Mg Coatings. Materials.

[29] Sun T., Jabar S., Kumar N., Liu C., Ceglarek D., Franciosa P. (2024). The impact of ring-shaped laser beam on dissimilar welding of Al-Cu thin sheets for battery tab-to-busbar connection: Microstructural, mechanical and electrical characteristics. Opt. Laser Technol..

